# Time–Motion Analysis by Playing Positions of Male Handball Players during the European Championship 2020

**DOI:** 10.3390/ijerph18062787

**Published:** 2021-03-10

**Authors:** Carmen Manchado, Basilio Pueo, Luis Javier Chirosa-Rios, Juan Tortosa-Martínez

**Affiliations:** 1 Physical Education and Sports, Faculty of Education, University of Alicante, 03690 San Vicente del Raspeig, Spain; carmen.manchado@ua.es (C.M.); juan.tortosa@ua.es (J.T.-M.); 2 European Handball Federation, Competitions Commission, 1120 Vienna, Austria; 3 Department of Physical Education and Sports, University of Granada, 18011 Granada, Spain; lchirosa@ugr.es

**Keywords:** covered distance, running pace, LPS, load monitoring, performance analysis

## Abstract

The aim of this study was to analyze time–motion characteristics of elite male handball players during the last European Championship 2020. A total of 414 players from 24 national teams were analyzed during 65 matches using a local positioning system (LPS) for the first time in a European Championship. Players (*n* = 1865) covered significantly (*p* < 0.001; ES = 0.48) more total distance in offense (1217.48 ± 699.33 m) and in all locomotion categories (*p* < 0.001) than in defense (900.96 ± 538.95 m), with a similar average total time on court (13.40 ± 8.19 min in offense and 13.27 ± 8.59 min; *p* > 0.05). The running pace was significantly higher in offense 96.53 ± 22.57 m/min than in defense 82.72 ± 43.28 m/min (*p* < 0.001; ES = 0.47). By playing positions, the Left Wing players covered significantly (*p* < 0.001) higher distances (2547.14 ± 1309.52) and showed longer playing time (32.08 ± 17.01). Center Back was the playing position that showed the highest global running pace (98.34 m/min). Players with higher running pace in offense (*p* < 0.001) were Left Backs (105.95 ± 25.20) and the Center Backs in defense (95.76 ± 48.90). There were no significant differences between winners and losers or between top ranked and lower ranked teams in terms of time played, distance covered, and running pace. Specific physical conditioning is necessary to maximize performance and minimize fatigue when performing in long tournaments.

## 1. Introduction

Handball is a professional and Olympic sport played worldwide that has become increasingly popular over the past decades. Two alternating sides characterize the game: the offense and the defense. The offense starts the moment the team gains possession of the ball and lasts until they lose it. During this period, the players try to score by employing individual, group, or team technical-tactical elements. The defense starts the moment the team loses possession of the ball and lasts until they regain it. During this period, the defenders try to obstruct the opponents’ scoring attempts by employing individual, group, or team technical-tactical elements [1].

Handball rules were modified in 2000 and 2016 and playing conditions adapted [2], which has increased the speed of the game (e.g., quick throw-off, goalkeeper as court player), making sport more marketable, enhancing media coverage, and making it more appealing for the viewer [3]. As a result of these rules changes, handball has increased in terms of dynamics, velocity, and intensity in the past years [4]. This is especially true for top-level handball.

Knowledge of the demands of the game is essential for the design of handball-specific training drills. Detailed information on the movements, like the distances covered by players, their movement velocities during a game—among the analysis of vertical movements like jumps, shots, or blocking—provides comprehensive assessment of the competition demands and assists in developing specific training regimes [5]. To be optimal, these training programs should be individualized with respect to playing positions and related to specific competition on-court demands [6].

This necessity to understand handball’s physical characteristics has raised great interest among researchers who have studied these demands using different methodologies [5]. The most widely used method has been time–motion analysis, based on observing players in the competition followed by an analysis of a video, taken with one camera [7,8] or two cameras [9]. The video-recorded matches are analyzed and the actions encoded. However, this method is time-consuming and depends on a subjective analysis of the observer, thus not being an objective or precise method when determining the different locomotion speeds.

In order to overcome this inconvenience, the European Handball Federation (EHF), Select^®^ and Kinexon^®^ jointly developed the Kinexon^®^ tracking system for handball players (Kinexon: München, Germany; Select Sport. Glostrup, Denmark) in addition to a monitored ball, the iball, which has been recently validated [10,11,12] and used in studies on handball [11,13] and other team sports [14]. This technology provides us with values regarding movements, accelerations, changes of direction, jumps, as well as data on the speed at which the ball is transferred (game speed) and the speed and position of the throws in real time, opening up new possibilities in the study of handball competition requirements [11]. With this fully automatic tracking system, the inconveniences mentioned for the conventional time–motion analysis are solved.

To date, there are no studies conducted with Local Positioning System (LPS) technology during a full championship of the highest level. There are also no studies at this level comparing the work done in both phases of the game, offense and defense. Therefore, new information must be provided through the use of this technology in high competition.

Previous time–motion studies carried out in different kinds of competitions, found differences between playing positions regarding the total distance covered and the distance covered at different locomotion categories [7,8,13,15]. Nevertheless, no studies so far have investigated in detail the activity profile of elite male handball throughout all matches of an European Championship for different playing positions. Therefore, the aim of the present study was to analyze on-court demands of handball players during the EHF European Championship 2020 to define time motion characteristics (time played, distance covered) both in offense and defense and according to their specific playing position, winners and losers, or between top ranked teams and lower ranked teams.

## 2. Materials and Methods

### 2.1. Subjects

Data were obtained from players participating in the European Handball Federation (EHF) EURO 2020, held in Austria/Norway/Sweden. Finally, 414 players were analyzed during the tournament. Goalkeepers were excluded from the analysis as distance and motion characteristics do not reflect their performance needs. Anthropometric characteristics and the age of the players are presented in Table 1. This information was collected from the official statistical data provided by the EHF.

### 2.2. Instruments

**Figure 1:**
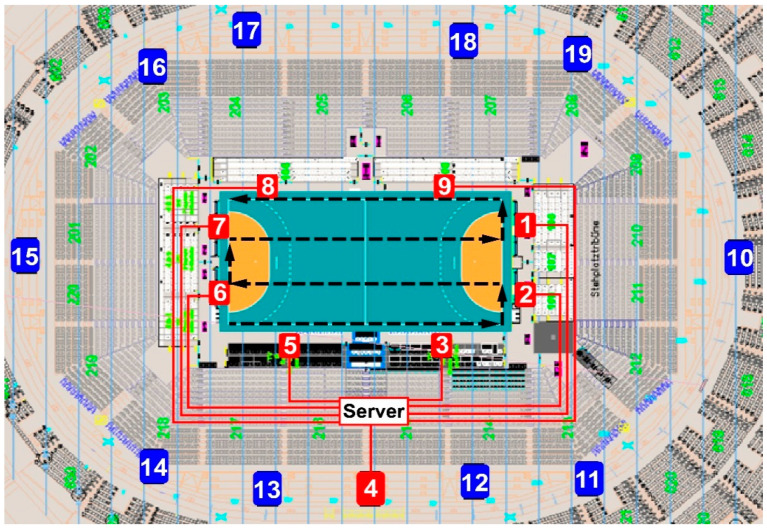
Local positioning system (LPS) setting: nine antennae connected to the server in red locations; ten reference antennae (anchors) in blue locations; meander path inside the field followed to check calibration accuracy (black discontinued line).

The players’ position data were collected through a LPS (Kinexon Precision Technologies, Munich, Germany), which has been recently validated [10,12] and used in studies on team sports [11,14], showing adequate between-device reliability (coefficient of variation around 5%) when compared to well-known systems such as GPS. Firmware versions and application software versions corresponded to the latest releases on the testing date (August 2019). Figure 1 shows the setting of the 9 antennae around the playing field, connected via Ethernet to the main server, and 10 anchor antennae distributed at 3 different levels above the ground in all EURO arenas.

UWB (ultra-wideband) LPS can be used as an alternative to professional optical systems and/or global satellite radio communication systems (GPS). In the context of indoor measurement, only camera-based technology can be used since GPS relies on direct paths between receivers carried by each player and a number of satellites. With LPS, the satellite-based measurement principle becomes local anchor-based measures using a local network of antennas, and therefore it can be used for both indoors and outdoors. LPS receivers usually include micro electro mechanical system (MEMs) to complement kinematic data retrieved by local positioning with individual player motion analysis. Typically, sensors include triaxial accelerometers measuring linear acceleration and gyroscopes, measuring angular velocity, all in the three coordinates. Unlike camera-based systems, the inertial sensors attached to each player allow for the precise body linear and angular kinematics to derive accelerations, jumps, impacts, and changes of direction.

In each venue, the LPS system were installed, calibrated, and checked for accuracy by a technician who worked for the manufacturer as follows. The exact position of the anchors in reference to the playing field was measured (blue numbered positions in Figure 1). Then, the anchor positions and the playing field position and size were transmitted to the Kinexon application. The location of one sensor at pre-defined positions (corner, penalty line, center point) was checked. In addition, two paths were followed to test the data quality and calculated distance—walking on the sideline and walking on a meander inside the field (black discontinued line in Figure 1). The devices worn by players comprised a sensor (player tag) positioned between the player’s shoulder blades using the manufacturer harness. The functionality of the sensors was tested in the venue by randomly walking and checking if signals were received from all units with adequate signal strength. These sensors transmit time signals via radio-technology to the antennae, which send signals via a wide local area network (WLAN) to local static base stations at known locations. A player’s momentary position is determined via 20 Hz frequency by calculating the time-of-flight (TOF) of ultra-wide-band radio signals traveling from the transmitter to the base stations, which calculate the actual 2D position of the devices within the playing field. Subsequently, instantaneous speed, i.e., scalar magnitude of velocity, as per the rate of change in horizontal x, y positions, and acceleration, as per the rate of change in speed, are derived by calculating the difference between two consecutive positions, i.e., approximating the derivative of the player’s position. The raw position and speed data are then filtered and smoothed by means of a Kalman filter for position data and an exponential moving average with a window length of 1 s for speed and position data. The system detects when a player is in possession of the ball, as it tracks both the players and the ball. Thus, it automatically changes offense to defense when appropriate. To this end, there was an automatic change from offensive to defensive for the team and vice versa at the moment where the ball possession changed. The respective offensive shift started with the ball possession of the team. Moreover, the system also checked if the players and the ball were moving in the direction of the opponent’s goal. In the event the ball was outside the court, the shift was interrupted. All data were analyzed using the system software (Kinexon Web Application, version 3.2.6, Munich, Germany).

### 2.3. Procedure

In this study, a descriptive observational cross-sectional study was used to examine the physical demands according to playing positions during competitive matches. This time–motion analysis has been used with team [16,17] and beach handball [18], as well as with other team sport studies [19,20].

The study was approved by the EHF. The participating teams signed an informed consent in the initial contract with the EHF to take part in the competition, where they accepted the rules and norms of the EHF, including their participation in different studies. The players’ data were anonymized for the purpose of this study. The players were informed of the purposes, procedures, and risks of the study and provided informed consent before the beginning of the study. All the procedures were conducted in accordance with the Declaration of Helsinki and approved by the Ethics Committee of the University of Alicante (registration number UA-2020-09-10).

The variables described next were measured based on position and speed data. The distances covered during the entire match (total distance/duration of play), distances per minute during play, and relative distance in established speed zones were computed. These zones were set as zone 1: standing (≤0.9 m/s), zone 2: walking (1.0–1.9 m/s), jogging (2.0–3.9 m/s), running (4.0–5.4 m/s), high-intensity running (5.5–6.9 m/s), and sprinting (≥7 m/s), in accordance with similar handball studies [7,8,21].

We also considered the distinction between offense (when the team was in possession of the ball) and defense (not in possession of the ball), and classified the players by their positions according to handball nomenclature in offense (left wing = LW, left back = LB, center back = CB; line player = LP; right back = RB; and right wing = RW).

### 2.4. Statistical Analysis

Descriptive analysis was presented as means and standard deviations. Data was analyzed for normality using the Kolmogorov–Smirnov test. The Mann-Whitney U test for independent samples was performed for analyzing the differences in playing time, distances covered, and running pace between offense and defense, winners and losers, as well as top and lower ranked teams.

The differences between the different playing positions in regards to playing time, distances covered, and running pace were determined by variance analysis one-way ANOVA, followed by Games–Howell post hoc testing, appropriate when there is a lack of homogeneity of variances. In order to determine the magnitude of each relationship, Cohen’s effect size (ES) was used with a modified classification (trivial <0.2, small 0.21–0.6, moderate 0.61–1.2, large 1.21–1.99, and very large >2.0) proposed for sports sciences [22] and used in other similar handball studies [13,21]. The alpha level of significance was set at *p* < 0.05. Statistical analysis was performed using the Statistical Package for Social Sciences (SPSS V22.0 for Windows, SPSS Inc, Chicago, IL, USA).

## 3. Results

### 3.1. Time on Court, Distance Covered, and Running Pace in Offense and Defense

**Figure 2:**
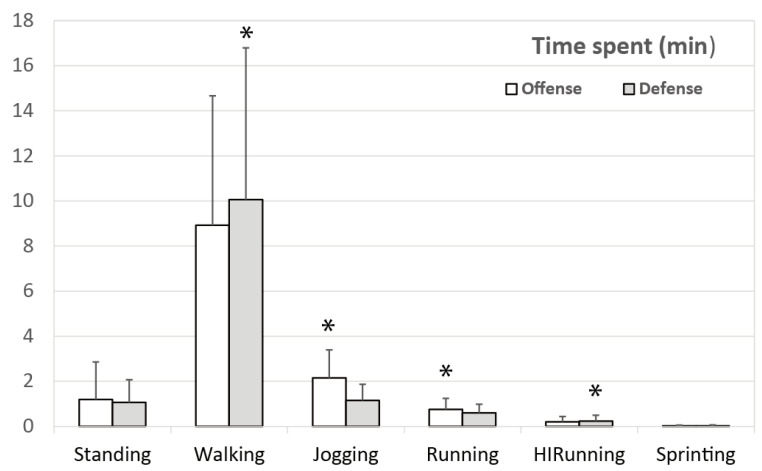
Time spent in court in each locomotion category by offense and defense. * Significant differences between offense and defense (*p* < 0.05).

The average time on court (*n* = 1865) during the European Championship was 26.48 ± 14.94 min, with 2.23 ± 2.35 min spent standing, 18.84 ± 10.64 min walking, 3.29 ± 1.73 min jogging, 1.35 ± 0.84 min running, 0.45 ± 0.47 min running at high intensity, and 0.03 ± 0.07 min sprinting. Significant differences were found between offense and defense (Figure 2) in time spent walking (8.92 ± 5.74 min vs. 10.06 ± 6.73 min; *p* < 0.001; ES = 0.19), jogging (2.15 ± 1.24 min vs. 1.15 ± 0.72; *p* < 0.001; ES = 0.89), running (0.75 ± 0.49 min vs. 0.60 ± 0.38 min; *p* < 0.001; ES = 0.32), and high intensity running (0.20 ± 0.24 min vs. 0.24 ± 0.25 min; *p* < 0.001; ES = 0.16). There were no significant differences for total played time (13.40 ± 8.19 min vs. 13.27 ± 8.59 min; *p* > 0.05) or time standing (1.19 ± 1.67 min vs. 1.06 ± 1.01; *p* > 0.05).

**Figure 3:**
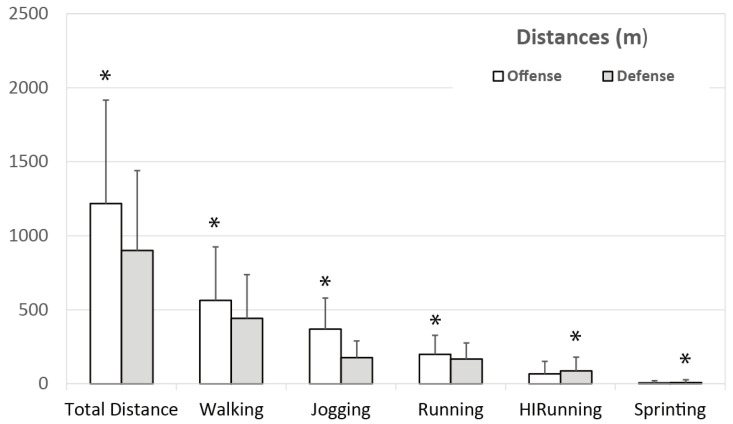
Distance covered in each locomotion category by offense and defense. * Significant differences between offense and defense (*p* < 0.05).

The average distance (*n* = 1865) covered during each game was 2105.78 ± 1150.76 m, with 999.95 ± 555.52 m covered walking, 544.32 ± 286.79 m jogging, 356.44 ± 225.67 m running, 153.59 ± 166.86 m running at high intensity, and 15.19 ± 30.80 m sprinting. Significant differences were found between offense and defense (Figure 3) in total distance covered (1217.48 ± 699.33 m vs. 900.96 ± 538.95 m; *p* < 0.001; ES = 0.48) as well as walking (564.03 ± 361.56 m vs. 442.12 ± 295.86 m; *p* < 0.001; ES = 0.35), jogging (370.01 ± 210.58 m vs. 176.87 ± 112.48 m; *p* < 0.001; ES = 1.02), running (190.99 ± 128.28 m vs. 167.47 ± 109.45; *p* < 0.001; ES = 0.19), high intensity running (67.31 ± 84.54 m vs. 87.64 ± 93.48 m; *p* < 0.001; ES = 0.23), and sprinting distances (6.34 ± 15.37 m vs. 9.07 ± 19.09 m; *p* < 0.001; ES = 0.17).

Locomotion activities were then normalized for each player according to the time they spent on court to obtain a true reflection of these demands, both for offense and defense. The total average running pace was 89.63 ± 35.18 m/min during a game. The running pace was significantly higher in offense 96.53 ± 22.57 m/min than in defense 82.72 ± 43.28 m/min (*p* < 0.001; ES = 0.47).

### 3.2. Time on Court, Distance Covered and Running Pace by Playing Positions

Table 2 shows the time played in the entire game according to different locomotion categories. Significant differences were found between the different playing positions in the total played time (F = 14.21; *p* < 0.001), time standing (F = 163.35; *p* < 0.001), walking (F = 4.94; *p* < 0.001), jogging (F = 15.13; *p* < 0.001), running (F = 36.91; *p* < 0.001), high intensity running (F = 245.57; *p* < 0.001), and sprinting (F = 237.50; *p* < 0.001).

Post-hoc analysis revealed that LWs played significantly more total time than the LBs (*p* < 0.001; ES = 0.57), CBs (*p* < 0.001; ES = 0.47), RBs (*p* < 0.001; ES = 0.50), and LPs (*p* < 0.001; ES = 0.47), but similar minutes than the RWs (*p* > 0.05). Similarly, RWs played significantly higher total time than LBs (*p* < 0.001; ES = 0.41), CBs (*p* < 0.001; ES = 0.31), RBs (*p* < 0.001; ES = 0.35) and LPs (*p* < 0.001; ES = 0.35). Left and RBs, CBs, and LPs played a similar average time (*p* > 0.05).

In regards to time standing, LWs and RWs spent longer time standing than the other playing positions but similar time between each other (*p* > 0.05). There were significant differences between the LWs and LBs (*p* < 0.001; ES = 1.41), CBs (*p* < 0.001; ES = 1.56), RBs (*p* < 0.001; ES = 1.45), and LPs (*p* < 0.001; ES = 1.28), while RWs also spent significantly longer time standing than the LBs (*p* < 0.001; ES = 1.27), CBs (*p* < 0.001; ES = 1.42), RBs (*p* < 0.001; ES = 1.31), and LPs (*p* < 0.001; ES = 1.13). The LP was the third position spending longer time standing, showing significant differences with the LB and RB (*p* = 0.001; ES = 0.30; and *p* < 0.001; ES = 0.42, respectively), as well as the CB (*p* < 0.001; ES = 0.63). The player position spending less time standing was the CB, with significant differences with the LB and RB (*p* = 0.004; ES = 0.29; and *p* = 0.039; ES = 0.08, respectively).

Results also showed that LWs spent significantly more time walking than LBs (*p* = 0.001; ES = 0.37), CBs (*p* = 0.007; ES = 0.32), RBs (*p* = 0.040; ES = 0.28), and LPs (*p* = 0.009; ES = 0.30). In regards to jogging time, the CBs were the players spending more time in this locomotion category showing significant differences with the rest of playing positions (LW, *p* < 0.001, ES = 0.46; RW, *p* < 0.001, ES = 0.63; RB, *p* < 0.001, ES = 0.42; LBs, *p* < 0.001, ES = 0.38; LPs, *p* < 0.001, ES = 0.64). LBs were the second playing position spending more time jogging showing significant differences with the RWs (*p* = 0.022; ES = 0.28) and LPs (*p* = 0.013; ES = 0.25).

LWs were the players spending more time running with significant differences with the rest of playing positions (CB, *p* < 0.001, ES = 0.62; RB, *p* < 0.001, ES = 0.79; LB, *p* < 0.001, ES = 0.88; LP, *p* < 0.001, ES = 0.76) except for RWs (*p* > 0.05). Similarly, RWs spent more time running than these paying positions (CB, *p* < 0.001, ES = 0.47; RB, *p* < 0.001, ES = 0.62; LB, *p* < 0.001, ES = 0.69; LP, *p* < 0.001, ES = 0.60). CBs also spent more time running than LBs (*p* = 0.032; ES = 0.27).

Again, LWs were the players spending significantly more time running at high intensity compared to the other playing positions (CB, *p* < 0.001, ES = 1.53; RB, *p* < 0.001, ES = 1.85; LB, *p* < 0.001, ES = 1.86; LP, *p* < 0.001, ES = 1.81) except for RWs (*p* > 0.05). Similarly, RWs spent more time running at high intensity than these playing positions (CB, *p* < 0.001, ES = 1.30; RB, *p* < 0.001, ES = 1.57; LB, *p* < 0.001, ES = 1.61; LP, *p* < 0.001, ES = 1.54). CBs were the third playing position spending more time at high intensity running showing significant differences with LBs and RBs (*p* < 0.001; ES = 0.45; and *p* < 0.001; ES = 0.58, respectively) but not with LPs (*p* > 0.05). LPs also spent significantly higher time in this locomotion category than LBs and RBs (*p* = 0.003; ES = 0.29; and *p* < 0.001; ES = 0.40, respectively).

Not surprisingly therefore, LWs spent significantly more time sprinting than the rest of playing positions (CB, *p* < 0.001, ES = 1.58; RB, *p* < 0.001, ES = 1.57; LB, *p* < 0.001, ES = 1.69; LP, *p* < 0.001, ES = 1.95) except for RWs (*p* > 0.05). Similarly, RWs spent more time running at high intensity than the rest of the players with exception to the LWs (CB, *p* < 0.001, ES = 1.27; RB, *p* < 0.001, ES = 1.27; LBs, *p* < 0.001, ES = 1.36; LP, *p* < 0.001, ES = 1.61).

In regards to distances covered (Table 3), significant differences were found for total distance covered (F = 17.61; *p* < 0.001), distance covered walking (F = 3.76; *p* = 0.002), jogging (F = 13.47; *p* < 0.001), running (F = 44.33; *p* < 0.001), at high intensity running (F = 256.67; *p* < 0.001) and sprinting (F = 234.04; *p* < 0.001).

**Figure 4:**
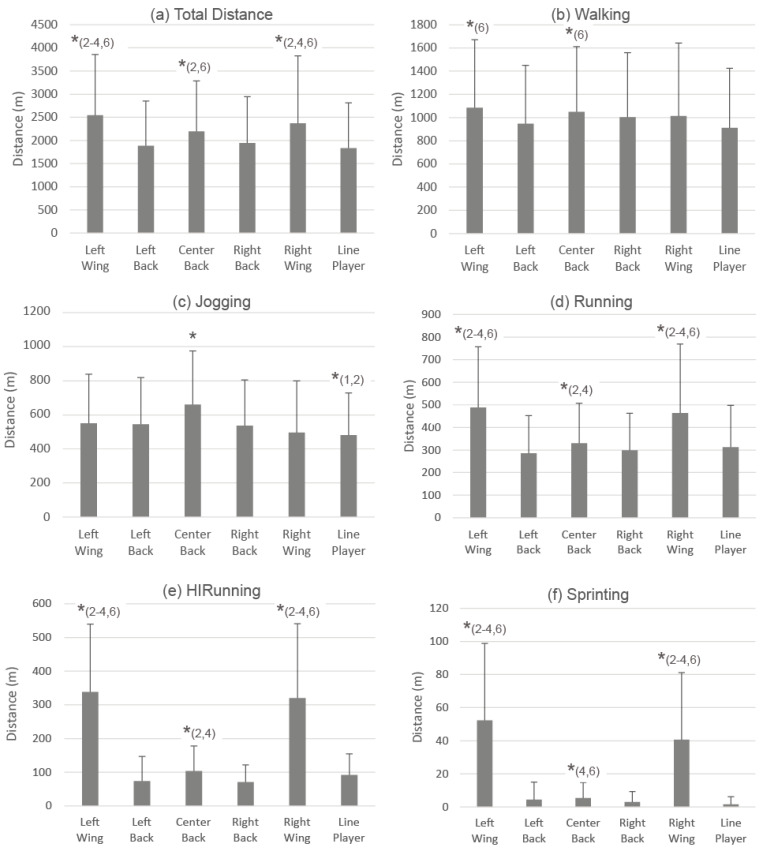
Distance covered in different locomotion categories by position. * Significant differences between positions indicated by numbers: 1 Left wing, 2 Left back, 3 Center back, 4 Right back, 5 Right wing, and 6 Line player.

Post-hoc analysis (Figure 4) showed that LWs were the ones covering a longer total distance compared to the other playing positions (CB, *p* = 0.017, ES = 0.29; RB, *p* < 0.001, ES = 0.54; LB, *p* < 0.001, ES = 0.59; LP, *p* < 0.001, ES = 0.64) except for RW (*p* > 0.05). RW also covered a longer total distance respect to the rest of playing positions (RB, *p* < 0.001, ES = 0.35; LB, *p* < 0.001, ES = 0.41; LP, *p* < 0.001, ES = 0.45), except for CB (*p* > 0.05). Next, CB covered a significantly longer total distance than LB (*p* = 0.011; ES = 0.30) and LP (*p* = 0.001; ES = 0.35).

LWs were the players covering a higher distance walking but showed significant differences only with LP (*p* = 0.005; ES = 0.32). CB covered the second higher distance showing significant differences with the LP (*p* = 0.035; ES = 0.26).

In regards to jogging, CB were the players covering a higher distance in this locomotion category showing significant differences with the rest of playing positions (LW, *p* < 0.001, ES = 0.37; RW, *p* = 0.002, ES = 0.53; Right backs, *p* < 0.001, ES = 0.43; LB, *p* < 0.001, ES = 0.40; LP, *p* < 0.001, ES = 0.65). LPs were the ones covering the lowest distance, showing significant differences with the left wings (*p* = 0.040, ES = 0.26) and LBs (*p* = 0.015, ES = 0.25).

LWs covered a significantly higher distance running than the rest of players (Center back, *p* < 0.001, ES = 0.71; RBs, *p* < 0.001, ES = 0.87; LBs, *p* < 0.001, ES = 0.95; LPs, *p* < 0.001, ES = 0.81) except for right wings (*p* > 0.05). RWs also covered a longer total distance respect to the rest of player’s positions (CB, *p* < 0.001, ES = 0.54; RB, *p* < 0.001, ES = 0.69; LB, *p* < 0.001, ES = 0.76; LP, *p* < 0.001, ES = 0.64). CBs were the third playing position covering higher distance running showing significant differences with LBs (*p* = 0.041; ES = 0.26).

LWs covered a significantly higher distance at high intensity running compared to the rest of players (CBs, *p* < 0.001, ES = 1.58; RBs, *p* < 0.001, ES = 1.87; LBs, *p* < 0.001, ES = 1.89; LPs, *p* < 0.001, ES = 1.87) except for RWs (*p* > 0.05). Similarly, RWs also covered a longer total distance respect to the rest of playing positions (CB, *p* < 0.001, ES = 1.33; RB, *p* < 0.001, ES = 1.58; LB, *p* < 0.001, ES = 1.60; LP, *p* < 0.001, ES = 1.46). Next, CBs covered significantly higher distances than RBs (*p* < 0.001; ES = 0.52) and LBs (*p* < 0.001, ES = 0.41) but not compared to LPs (*p* > 0.05).

LWs covered a significantly higher distance sprinting than the rest of players (CBs, *p* < 0.001, ES = 1.45; RBs, *p* < 0.001, ES = 1.54; LBs, *p* < 0.001, ES = 1.57; LPs, *p* < 0.001, ES = 1.64) other than the RWs (*p* > 0.05). Similarly, RWs also covered a longer total distance respect to the rest of player positions (CB, *p* < 0.001, ES = 1.22; RB, *p* < 0.001, ES = 1.33; LB, *p* < 0.001, ES = 1.33; LPs, *p* < 0.001, ES = 1.42). CBs present the third highest covered distance showing significant differences with RBs (*p* < 0.014; ES = 0.30) and LPs (*p* < 0.001; ES = 0.54).

Table 4 shows the running pace in the entire game, offense, and defense according to each player position. The ANOVA showed significant differences in the running pace between the different playing positions (F = 11.52; *p* < 0.001). Post-hoc analysis revealed that the players with a higher running pace were the CBs with significant differences with the rest of the playing positions (LW, *p* < 0.001, ES = 0.48; RW, *p* < 0.001, ES = 0.39; RB, *p* < 0.001, ES = 0.35; LB, *p* < 0.001, ES = 0.21; LP, *p* < 0.001, ES = 0.18). LPs were the second playing position with a higher running pace showing significant differences with the LWs and RWs (*p* = 0.001, ES = 0.20; *p* = 0.047, ES = 0.16, respectively), but not with the LBs and RBs (*p* > 0.05). LBs showed the third highest running pace with significant differences found with the LWs and RWs (*p* = 0.001, ES = 0.23; *p* = 0.048, ES = 0.17, respectively). No significant differences were obtained between the RBs and the wings players (*p* > 0.05).

In offense, significant differences were obtained for the different playing positions (F = 25.26; *p* < 0.001). Players with a higher running pace in offense were LBs, showing significant differences with the rest of the playing positions (LW, *p* < 0.001, ES = 0.67; RW, *p* < 0.001, ES = 0.75; RB, *p* < 0.001, ES = 0.53; CB, *p* < 0.001, ES = 0.23; LP, *p* < 0.001, ES = 0.43). CBs had the second highest running pace with significant differences with the LWs and RWs (*p* = 0.001, ES = 0.61; *p* = 0.048, ES = 0.65, respectively) and a trend with the LP (*p* = 0.053, ES = 0.30). Next RBs, showing significant differences with RWs (*p* < 0.001, ES = 0.36), but not with LWs or the LPs (*p* > 0.05). RWs showed significant differences with LPs (*p* < 0.001, ES = 0.16), but not with LWs (*p* > 0.05).

In defense, the ANOVA also revealed significant differences for the different playing positions (F = 8.12; *p* < 0.001). CBs were the ones with the highest running pace showing significant differences with the rest of players (LWs, *p* < 0.001, ES = 0.48; RWs, *p* = 0.018, ES = 0.29; RBs, *p* < 0.001, ES = 0.39; LBs, *p* < 0.001, ES = 0.47) except with LPs (*p* > 0.05). LPs had the second highest running pace with significant differences with LWs (*p* = 0.028, ES = 0.23), and LBs (*p* = 0.021, ES = 0.24) but not with CBs, RBs, and RWs (*p* > 0.05).

### 3.3. Differences by Winners and Losers and between Top and Lower Ranked Teams

There were no significant differences between winners and losers or between top ranked teams and lower ranked teams in regards to total time played, distance covered, and running pace. In offense, there were significant differences between teams according to their rank in the championship in time standing (F = 4.469; *p* = 0.012) and time running (F = 4.778; *p* = 0.009). Post-hoc analysis showed significant differences for time running between the teams ranked 1–6 and 7–12 (*p* = 0.025; ES = 0.17) and between teams ranked 1–6 respect to teams 13-24 (*p* = 0.023; ES = 0.15). Significant differences were also found in offense between teams according to their rank in the championship in distance running (F = 4.951; *p* = 0.006). Post-hoc analysis showed significant differences for distance running between the teams ranked 1–6 and 7–12 (*p* = 0.026; ES = 0.17) and between teams ranked 1–6 respect to teams 13–24 (*p* = 0.017; ES = 0.16). In defense, no significant differences were found between winners and losers or between top ranked teams and lower ranked teams in either time played, distances covered, or running pace.

## 4. Discussion

The main aim of this study was to examine the differences in time spent on the court and distance covered in a male European Handball Championship, taking into account the influence of the offense-defense game phases. To our knowledge, it is the first study to analyze such a championship, taking into account offense and defense. It is also a novelty because of the large number of matches and players monitored and for the technology used.

### 4.1. Time on Court, Distance Covered, and Running Pace in Offense and Defense

The absolute data concerning total distance covered and total time played in our study show lower values than previously reported by Manchado et al. [13] (15% less played time and 22% less covered distance), although the same LPS technology has been used and top-level European players are analyzed. The difference can be due to the number of matches and the type of competition. In their study, these researchers analyzed the VELUX EHF FINAL4 2019 for clubs, in which few matches were played. In the present study a longer tournament for National Teams, played in a very short period of time, is analyzed. It is therefore a more demanding competition, as a result coaches tended to make more substitutions to maintain performance.

The data from our study in this regard are also 28% lower in played time and 19% lower in covered distance from the average values reported by Cardinale et al. [21], in its analysis of the World Championship 2015 (36.48 ± 20.27 min and 2607.5 m). Although high-level National Teams were analyzed as well, a different system was used (automatic tracking with cameras). In addition, a better performance level of players from European teams, will probably favor a better dosage of the minutes of play between them. These two facts may account for the differences.

Other studies in European handball players carried out throughout a national league championship, reported data even more different from ours, above 40 min [7,8,23]. The explanation, in this case, may be due to the type of instruments used (video tracking) and the kind of competition (clubs). A recent review by Gómez-Carmona et al. [24] on the use of technologies in external load control in team sports concluded that LPS systems are more accurate than video tracking, which may explain some of the differences found.

The results, focusing on the analyzed locomotion categories, are consistent with most of the handball time–motion studies, indicating during a significant duration of the game (over 70%), players perform low-intensity activities [7,9,13,21,23], walking, jogging, running, and only about 10% of the locomotion is done at high speed, which confirms that handball is an intermittent sport in which high intensity activities are the key to performance [8,25,26,27] and that high percentage of low intensity loads enable an athlete profile with medium values in aerobic capacity [28]. This should be considered by coaches for adequate training plans.

One of the main findings of the study is the significant differences found between offense and defense in the distances covered. The total covered distance in offense was 16% higher than in defense. However, the data indicate that players in defense covered a greater distance in high-intensity zones (23% in high intensity running and 30% in sprinting) than in offense. This implies from a physical point of view, that the game demands are different depending on whether the player takes on an offense or defense role.

These data are even more relevant if players remain on the court for similar time in both phases of the game, with no significant differences in total played time (50.6% of the played time in offense vs. 49.4% in defense). These results are consistent with those obtained by Manchado et al. [13], who also showed significant differences between offense and defense, with greater high intensity values for the defense. Thus, this fact should be taken into account by coaches when planning their programs.

Michalsik et al. [7] showed similar global percentages than those obtained by our study in high intensity zones. The differences appear when analyzing both phases of the game. Compared with the present study, a higher percentage of high intensity activities (HI running+ sprinting) was reported in offense (8.8% vs. 6.1%) and a lower percentage in the defense (7.0% vs. 10.73%). These differences can also be mediated by the methodology (manual tracking), the instrumental (video camera), and the sample used (two teams monitored during an entire league competition).

These are the only studies we have found that analyze the differences between offense and defense in professional players. There are other works, but not with high level players or not analyzing defensive playing positions [17,21,29]. Thus, more studies are needed that delve deeper into the differences in physical requirements between offense and defense.

In order to facilitate the comparison between studies and to improve comprehension for coaches by having an easier-to-understand load index, data were normalized in m/min. Our work shows global values of 89.63 ± 35.32 m/min, being significantly higher (*p* < 0.001; ES = 0.47) the running pace for the offense than for the defense (14.3% higher). However, the defense still has a higher relative index in the most intense zones. Manchado et al. [13], using LPS technology, reported similar data 86.90 m/min (Off = 88.45 ± 20.72 m/min, Def = 80.83 ± 27.11 m/min), also finding significant differences between offense and defense, with greater values in the high intensity zones for the defense. However, the overall data reported by Cardinale et al. [21] in its analysis of the World Championship 2015 are clearly below (74.21 m/min). It is a similar competition but in which there are lower-demanding matches, because of the disparity in levels, which may explain these differences. In the EURO, the team’s performance level is more homogeneous.

### 4.2. Time on Court, Distance Covered, and Running Pace by Playing Positions

As a second goal, it was intended to examine how playing position affects the variables. To the best of our knowledge, this is the first study to provide detailed information of elite male handball players on-court demands based on the different playing positions in offence and defense during a EURO.

The analysis of the different playing positions, reported absolute and relative differences in the on-court activities that players perform depending on the specific position. The wing players are those that covered significantly higher distances and showed longer played time (about 500 meters more than average and 7 minutes more on the court). The data support the results found by other research in this same line [13,21,23]. In addition, the wings are the players who significantly covered a greater distance at high intensity compared to the other teammates (approx. 300 m more at high intensity running and sprinting). These data are in line with other studies that showed similar differences between the wings and the other court players [7,13,17,21].

By normalizing the data according to the played time, the CBs were clearly the playing position that showed the highest running pace (98.34 m/min). Interestingly, LP is the second playing position who showed higher relative intensity, even being above the LWs and RWs players, positions that showed significantly lower Running pace with exception to the RBs players. This normalized data has to be taken into account by coaches when planning their training.

One of the main findings of our study when comparing the different playing positions in offense and defense is that there are significant differences (*p* < 0.001) between them, once the data were normalized. It is interesting for coaches to know that in offense, LBs players are those who play at a significantly (*p* < 0.001) higher intensity than the rest of their teammates. In defense, the different specific positions also worked differently. In this case, the CBs play at a significantly higher intensity than the rest except the LPs, which are the seconds that work at the most intensity. The results are consistent with those of Manchado et al. [13] in which CBs and Front Center Defenders worked significantly at greater intensity than the rest of the playing positions. In handball, players are quite specialized to a specific position. It is true that during the match, some players transform their position (wing-to-pivot or back), but do so temporarily, getting back to their specific position. Only Back players (right, left, and center) exchange their positions on a regular basis. That is the reason why we believe it is also necessary to individualize their training programs, not only at the technical-tactical level but also at the conditional level. Our findings confirm studies in the same line [23], to which it should be added that this difference must be analyzed both in offense and in defense, because as it has been shown, demands are different for each position and phase of the game.

### 4.3. Differences by Winners and Losers and between Top and Lower Ranked Teams

One of the issues to be highlighted is that the global playing time and player’s distances covered, were neither different for winners or losers, nor affected by the final ranking. These data are in consonance with those obtained by Cardinale et al. [21]. This may be because, at this level of performance where all the players are physically well developed, playing time and distance covered are not differentiating variables. The weight of these differences may be related to other variables such as the tactical component or anthropometric differences, among others. More studies are needed in this regard to try to ascertain which variables are the most influential to differentiate one winning team from another.

This study is the first to analyze a top-level championship using LPS microsensors. This system has proven to be more accurate for evaluating distances than video systems [24] and therefore coaches and researchers could consider these results as a reference. With the current technological advances, it would be necessary to rethink the categories established to assess the physical requirements existing in handball game [13,30,31]. The procedures used so far are not well defined. Categories have been obtained indirectly [9,17,21] or based on other sports [7,8,32,33] and may not reflect the reality of the game, dismissing the maximum efforts. It is necessary to find formulas, with external and internal control of the load using microsensors, that better represent the requirements of the competition, so studies in this regard are necessary.

This study presents some limitations. The main one is that only one men’s European championship is analyzed, although many matches and all players have been analyzed. There are also currently few studies that have analyzed high-level handball competitions using LPS technology, which makes it difficult to compare the results. Therefore, more research is needed in this regard that provides coaches with objective competition data, which are extrapolable to training. The analysis should also address similar studies in other categories, both male and female, in order to establish benchmarks for researchers and coaches.

A recent review [24] clearly indicated the benefit of LPS over other systems such as the widely used video-based systems. One of the main advantages of LPS is that they can offer accelerometry data including accelerations, jumps, impacts, or changes of direction, an aspect for which video-based systems are not technologically prepared. However, LPS systems are quite expensive so only the Bundesliga and some clubs, such as PSG or FC Barcelona, and some National Federations (e.g., Norway, the Netherlands, and France) have LPS systems in their facilities. Thus, video recording remains the only possible resource for many entities. Future studies comparing both systems would be necessary to determine the pros and cons of each system.

Finally, although the data have been analyzed based on the two phases of the game (offense and defense), specific positions in defense have not been defined, as the data has been provided by KINEXON based on the offensive playing position.

## 5. Conclusions

Our findings suggest that coaches need to specifically differentiate the training load for each playing position, both in offense and in defense. The player’s profile will therefore be mediatized by the playing position on the field and consequently training must be individualized in consonance. The best way to control the load is through the running pace that, with other load indicators should serve as guidance for the coach to know the evolution of his players and as a method of assessing the specific player physical condition.

This study has addressed the analysis of time and distance, with reference to specific positions and phases of the game. In further studies, aspects related to the differences concerning activity patterns over the game (first and second half), or in specific minutes with great importance in the final result, could be faced. In addition, it would be of great interest to combine the study of internal and external load including more variables than this new technology allows, such as accelerations, jumps, and changes of direction.

## 6. Practical Applications

In order to design appropriate training programs for elite handball players, it is essential to know the real demands of the competition. Coaches should be able to know the different demands according to specific positions and phases of the game in order to individualize training loads.

This type of study should be used to control the task’s intensity level required during training practice. Our findings suggest that training strategies should consider position-specific exercises in order to improve the players’ ability to maintain high intensities during the entire match. In this regard, it would be interesting to control training loads and physical performance during training practice with the same or a similar system than the one used in competition, in order to obtain more comparable and transferable data.

## Figures and Tables

**Table 1:** Physical characteristics of the players (mean ± standard deviation).

Playing Position	*n*	Height (cm)	Body Mass (kg)	BMI (kg/m^2^)	Age (Years)
Left Wing	48	186.9 ± 5.7	84.4 ± 7.9	24.2	28.3 ± 4.6
Left Back	73	196.1 ± 4.2	97.2 ± 6.5	25.3	26.8 ± 4.7
Center Back	55	189.7 ± 5.8	90.3 ± 6.9	25.1	27.5 ± 5.0
Right Back	52	194.4 ± 5.8	95.7 ± 8.9	25.3	27.9 ± 4.8
Right Wing	50	184.6 ± 5.4	83.1 ± 6.3	24.4	28.0 ± 4.4
Line Player	79	196.8 ± 4.6	105.3 ± 8.5	27.2	28.5 ± 4.7
Total	414	192.4 ± 6.7	94.3 ± 10.5	25.5	28.1 ± 4.8

**Table 2:** Total time (minutes) played in different locomotion categories by playing positions.

	Left Wing	Left Back	Center Back	Right Back	Right Wing	Line Player
*n*	213	320	248	246	230	374
Total Time	32.08 ± 17.01	23.77 ± 12.58	24.91 ± 13.64	24.46 ± 13.30	29.98 ± 18.43	24.53 ± 13.81
Standing	4.57 ± 3.18	1.37 ± 1.36	1.02 ± 0.98	1.27 ± 0.98	4.22 ± 3.08	1.77 ± 1.31
Walking	21.23 ± 11.30	17.44 ± 9.43	17.85 ± 10.25	18.26 ± 10.29	19.98 ± 12.33	18.05 ± 10.40
Jogging	3.22 ± 1.71	3.36 ± 1.69	4.04 ± 1.91	3.29 ± 1.65	2.88 ± 1.76	2.96 ± 1.52
Running	1.81 ± 0.99	1.11 ± 0.64	1.29 ± 0.68	1.16 ± 0.64	1.72 ± 1.13	1.19 ± 0.70
HIRunning	0.97 ± 0.58	0.22 ± 0.22	0.32 ± 0.22	0.21 ± 0.15	0.93 ± 0.64	0.28 ± 0.19
Sprinting	0.13 ± 0.11	0.01 ± 0.02	0.01 ± 0.02	0.01 ± 0.02	0.10 ± 0.10	0.00 ± 0.01

**Table 3:** Total distance covered in different locomotion categories by playing positions.

	Left Wing	Left Back	Center Back	Right Back	Right Wing	Line Player
*n*	213	320	248	246	230	374
Total Distance	2547.14 ± 1309.52	1887.90 ± 962.22	2194.39 ± 1093.97	1943.25 ± 1003.03	2371.89 ± 1456.80	1835.23 ± 979.15
Walking	1083.56 ± 586.68	945.81 ± 505.53	1049.13 ± 561.89	1002.57 ± 557.20	1013.69 ± 629.55	912.11 ± 512.94
Jogging	550.19 ± 290.46	545.04 ± 274.35	661.30 ± 314.51	536.77 ± 268.38	495.91 ± 304.41	480.67 ± 248.13
Running	489.65 ± 268.09	285.83 ± 167.70	330.35 ± 177.98	297.88 ± 165.59	464.45 ± 304.97	312.72 ± 185.30
HIRunning	337.86 ± 202.40	73.79 ± 73.74	103.93 ± 74.43	70.78 ± 50.92	320.48 ± 221.36	92.07 ± 62.90
Sprinting	52.16 ± 46.48	4.32 ± 10.66	5.37 ± 9.36	2.95 ± 6.33	40.70 ± 40.46	1.47 ± 4.68

**Table 4:** Running pace (m/min) in the entire game, offense, and defense.

	Total	Offense	Defense
Left Wings	83.68 ± 23.50	90.65 ± 18.28	76.56 ± 25.96
Left Backs	90.83 ± 35.49	105.95 ± 25.20	75.67 ± 37.80
Center backs	98.34 ± 36.11	100.98 ± 15.48	95.76 ± 48.90
Right backs	86.52 ± 30.16	94.12 ± 18.04	78.92 ± 37.17
Right wings	85.12 ± 32.92	87.74 ± 12.32	82.19 ± 43.12
Line players	91.17 ± 42.67	95.84 ± 29.38	86.48 ± 52.39
Total	89.63 ± 35.32	96.53 ± 22.57	82.72 ± 43.28

## Data Availability

Restrictions apply to the availability of these data. Data was obtained from the European Handball Federation.
